# Assisting role redesign: a qualitative evaluation of the implementation of a podiatry assistant role to a community health setting utilising a traineeship approach

**DOI:** 10.1186/1757-1146-5-30

**Published:** 2012-11-27

**Authors:** Anna M Moran, Rosalie A Boyce, Alan M Borthwick, Karen Murphy

**Affiliations:** 1 Charles Sturt University, Albury, Australia; 2 Southern Cross University, Lismore, Australia; 3 ACT Government Health Directorate, Canberra, Australia; 4 University of Southern Queensland, Toowoomba, Queensland, Australia; 5 University of Southampton, Southhampton, UK

## Abstract

**Background:**

Increasing demands for podiatry combined with workforce shortages due to attrition, part-time working practices and rural healthcare shortages means that in some geographic areas in Australia there are insufficient professionals to meet service demand. Although podiatry assistants have been introduced to help relieve workforce shortages there has been little evaluation of their impact on patient, staff and/or service outcomes. This research explores the processes and outcomes of a ‘trainee’ approach to introducing a podiatry assistant (PA) role to a community setting in the Australian Capital Territory (ACT) Government Health Service Directorate.

**Method:**

A qualitative methodology was employed involving interviews and focus groups with service managers, qualified practitioners, the assistant, service users and consumer representatives. Perspectives of the implementation process; the traineeship approach; the underlying mechanisms that help or hinder the implementation process; and the perceived impact of the role were explored. Data were analysed using the Richie and Spencer Framework approach.

**Results:**

Although the impact of the PA role had not been measured at the time of the evaluation, the implementation of the PA traineeship was considered a success in terms of enabling the transfer of a basic foot-care service from nursing back to podiatry; releasing Enrolled Nurses (ENs) from foot-care duties; an increase in the number of treatments delivered by the podiatry service; and high levels of stakeholder satisfaction with the role. It was perceived that the transfer of the basic foot-care role from nursing to podiatry through the use of a PA impacted on communication and feedback loops between the PA and the podiatry service; the nursing-podiatry relationship; clinical governance around the foot-care service; and continuity of care for clients through the podiatry service. The traineeship was considered successful in terms of producing a PA whose skills were shaped by and directly met the needs of the practitioners with whom they worked. However, the resource intensiveness of the traineeship model was acknowledged by most who participated in the programme.

**Conclusions:**

This research has demonstrated that the implementation of a PA using a traineeship approach requires good coordination and communication with a number of agencies and staff and substantial resources to support training and supervision. There are added benefits of the new role to the podiatry service in terms of regaining control over podiatric services which was perceived to improve clinical governance and patient pathways.

## Background

Demand for allied health professionals in Australia is increasing due to the ageing population [1,2], increasing rates of chronic illness, and increasing recognition of the role that allied health providers can play in management of many long term conditions [3]. One of the implications of this change is an upward pressure on health care providers to focus on the more specialised areas of their job, while delegating the less skilled components to other staff [4,5].

In Australia, podiatry is a female dominated workforce (61%), with a high proportion of part-time workers and around one quarter of the workforce (25.7%) are employed full-time [6]. Health workforces characterised by high numbers of part-time workers and attrition rates require innovative retention strategies [3].

Public sector services are experiencing growing demand for treatment of patients with high risk podiatric issues and complex care requirements [3], thus reducing the capacity of podiatrists to offer basic foot-care to those at lower risk of complications. This situation is expected to worsen as the population ages and the incidence of chronic illness increases [2].

Although there is evidence of the introduction of podiatry support workers as early as 1977 [7,8] there has been little formal evaluation of their impact on the outcomes for patients, staff or services. There is evidence that the introduction of the role of qualified podiatry assistants has met with some resistance from the professional body in the UK [8].

Podiatry assistants are a relatively new concept in Australia and there have been diverse assistant ‘training’ and roles in existence, such as the ‘chairside’ assistants. The Podiatry Board of Australia defines podiatric assistants (PA) as *“staff employed within a facility or practice who is not a registered podiatrist and who assists a podiatrist in the delivery of services to his or her patients or clients”* p2 [9].

The delegation of tasks to qualified PAs has been shown to increase accessibility to foot-care services for targeted client groups by reducing waiting times for low risk procedures and increasing qualified podiatry hours [10].

Research conducted in the United Kingdom (UK) into the prevalence and type of foot conditions in the National Health Service (NHS) suggests that assistant practitioners in podiatry could provide a greater proportion of ‘core’ podiatry work [11], where ‘core podiatry work’ consists of treatment of the nails, corns and callus and also giving footwear and foot health advice [12]. This workforce role reform could feasibly translate into an Australian setting [13].

Another UK based study of podiatry staffing found that PAs were more likely to be employed in services with large numbers of podiatrists and more senior podiatrists [7]. The same study found that foot-care assistants perceived they had limited career advancement and that acquisition of competencies did not necessarily translate to higher pay unless they became registered practitioners. Furthermore a lack of national standards and localised employer based training and poor access to education structures and supervision meant that roles tended to be extremely varied and context specific and as such not easily transferable to other settings/services [14].

Research examining podiatrists' perceptions of professional status suggests that innovative measures, such as utilisation of PAs, is important to reduce the profession’s vulnerability to boundary encroachment by other professions and roles (e.g. nurses and foot-care assistants) [15].

The Australasian Podiatry Council (APodC) guidelines stipulate that PAs can provide “direct practical support to the podiatrist” and there is “a role for a suitably trained assistant providing foot hygiene services to patients identified by the supervising podiatrist as requiring this service” [16]. The APodC’s *Policy on Podiatry Assistants* also mentions that “in terms of clinical care and the use of instrumentation, hands-on foot-care provided by a podiatry assistant is in the form of foot hygiene only and is to be conducted on low-risk patients”. As such these regulations are a driving force in defining roles, role variation and role boundaries within podiatry [5].

Various models of allied health assistant training exist [17], although there is no systematic overview of these. Most approaches incorporate varying levels of on-the-job training and formal training to achieve specific competencies. Other models of therapy assistants training involve cycles of observation, practice under observation until competency is reached, followed by on-going documentation and supervision [18]. There is also evidence to suggest that specific local requirements of the role need to be balanced against the drive to achieve consistency and uniformity in AHA training [19].

Two studies illustrate the limitations of training assistants, away from the workplace, without the involvement of staff that will be working with the new AHA [20,21]. Both found that supervising staff were reluctant to delegate tasks to the assistant practitioners because they did not understand the competencies or experience that they brought to the job. This resulted in inconsistent use of the assistants, the underutilisation of their skills and abilities, and some dissatisfaction with their work as a result.

The use of allied health assistants in Australia is fast becoming a way to meet the future health and social care demands from a growing and ageing population and to account for a shortfall in professionally qualified practitioners [3,4]. Yet evidence examining the way this may come about is lacking.

A large metropolitan health service in the Australian Capital Territory (ACT) identified a need to introduce Certificate IV trained podiatry assistants into community health settings and as such recently introduced a new podiatry assistant role into one community health service. The role was implemented using a traineeship approach, where an unqualified worker undertakes a combination of study with a Registered Training Organisation (RTO) plus on-the-job training to achieve competency in the Certificate IV in Allied Health Assistance, including all competencies in the Podiatry skillset.

The results of an evaluation of the processes and outcomes associated with implementing a new traineeship PA role to a community health setting is presented here.

### Background to the implementation of the podiatry assistant role

Within the Australian Capital Territory (ACT) Government Health Service Directorate, unmet need for basic foot-care was met by enrolled nurses (ENs) who had undergone foot-care-specific training provided by in-house podiatrists [22]. This role was limited to the provision of basic foot-care. The Foot-care Nurse (FCN) service was provided by ENs from the Community Nursing team, which was organisationally separate to the podiatry service.

The Australian Capital Territory (ACT) first introduced AHAs in 2004 and in 2005, formal training at Certificate IV level was introduced by the local Registered Training Organisation (RTO) in Physiotherapy, Speech Pathology and Occupational Therapy. This was later supported by a national health training framework in Allied Health Assistance developed by the Industry Skills Council [23].

In 2009, infrastructure investment funding was made available in the ACT to develop new health professional support roles. The ACT Government Health Directorate obtained funding for the development of 7 new allied health assistant roles over 3 financial years. Within this, two new allied health assistant traineeship roles were introduced in 2010 (in speech-language pathology and podiatry) with a further 5 taken on in 2011.

Podiatrists and managers hoped that an appropriately qualified podiatry assistant, managed by the podiatry team, would enable the service to co-ordinate the full range of foot treatment services as the current ‘low risk’ foot-care service was operated, managed and conducted by nursing. This model was proposed to offer optimal patient care and a more seamless patient journey when clinical needs transitioned from basic to complex.

A dedicated podiatry assistant (PA) was trained to Certificate IV level to fill these specific service gaps as well as to provide a more accessible and better quality service for people with basic foot-care needs. It was also hoped that the PA would eventually be able to assist in other activities such as health promotion, nail surgery and making orthotic modifications under the prescription of the podiatrist.

## Methods

A qualitative methodology was employed to explore service manager, qualified practitioner, assistant and service user perspectives of: the implementation process; the traineeship approach; the underlying mechanisms that help or hinder the implementation, sustainability and impact of the role; and the perceived impact of the role. As such the research questions for this study are;

1. What mechanisms are required to implement a new traineeship assistant role in podiatry to a community health setting and;

2. What is the perceived impact of the role and traineeship approach?

Data collection involved interviews, focus groups and documentary analysis of job descriptions, competency frameworks and policy documents. The focus group and individual interview schedules were constructed by AM and SN, informed by results from a recent literature review [14] and previous research examining implementation of new roles [21] and podiatry assistants [7,10]. Table 1 details the topic schedules for the interviews and focus groups. A purposive sampling method was used to ensure representation within each of the following groups:

Group 1 the PA (n = 1) [individual interview];

Group 2 the qualified podiatrists who work with and supervise the PA (n = 3) [focus group];

Group 3 the enrolled nurses who who had been performing foot-care for the health service (n = 3) [focus group];

Group 4 directors of allied health, service managers and lead professional and clinical educator of the service (podiatry) (n = 5) [individual interviews & focus group]; and

Group 5 service users from community care podiatry services (n = 5) [focus group] and members of a consumer group (n = 3) [focus group].

**Table 1:** Interview and focus group schedules explored the following issues

**Service users and the consumer group**	**Allied Health managers, nursing managers and podiatrists**	**Enrolled Nurses**	**Trainee PA**
· Exploration of their use of the service	· Exploration of their service	· Background to their role in foot-care	· Exploration of their service
· Exploration of their interface with the podiatrist and trainee PA	· Why podiatry assistants were introduced	· How their role differs to the PA role	· Their background before training as a PA
· General feelings about the use of assistants	· The impact of the introduction of the assistant on their role, workload, patient throughput (etc.)	· How the PA role was introduced	· What attracted them to the role
		· Relationship to and with the new PA	· Why the (PA) role was introduced
· Understanding of, and preferences for levels of training of Pas			
		· he impact of the PA on their role and their service	· Their training background (including traineeship)
· Important qualities of PAs			
	· Relationship with the PA	· Mechanisms that help/hinder the new role to be effective	· Mechanisms that help/hinder them to be effective in the new role
	· Understanding of the supervision support		
			· How is it decided what roles/duties they undertake in their service
	· Governance/responsibility	· Issues that have arisen with the new role	
	· Current and other possible ways to measure the PA’s effectiveness		· Relationship with qualified practitioners (delegation of roles, autonomy, responsibility)
	· Effectiveness of the implementation process		
	· Mechanisms that help/hinder the PA to be effective		· Supervision arrangements
			· Career development opportunities
	· Decisions around roles/duties undertaken		
	· How the PA workload is determined (role boundaries)		

### Recruitment

Staff were invited to participate in the study *via* a letter of invitation outlining the study with the opportunity to volunteer to participate in a focus group session/interview by contacting a nominated person. Informed consent was attained prior to conducting the interviews. Service users were informed of the study in the first instance by staff at the community based service they attended *via* a letter of invitation outlining the study. The letter provided patients with the opportunity to volunteer to participate in a focus group session by contacting a nominated person who then posted an information sheet.

### Analysis

The focus groups and interviews were recorded and transcribed verbatim. The Ritchie and Spencer qualitative *Framework* approach was used as the analytical framework [24]. This involves familiarisation with the data; identifying a thematic framework; indexing the themes; charting those themes into a hierarchical framework; then mapping and then interpretation of those themes [24]. A coding framework was established based on *a priori* issues which formed the basis of the research questions and interview schedule. An initial coding template was then developed using the *in vivo* terms used by interviewees (Table 2). The resulting coding framework was then hierarchically organised under the dominant themes identified from the combined focus group and interview data. Key information extracted from policy and competency documents was then utilised to clarify and/or reiterate findings from the interview and focus group data.

**Table 2:** Initial coding template

· The implementation of the podiatry assistant role in podiatry/why the role was introduced
· Traineeship model
· The roles and responsibilities of the podiatry assistant (scope of practice)
· What makes a good podiatry assistant?
· Negotiation of roles
· Supervision and support structures
· Factors that facilitated the introduction of the podiatry assistant role
· Barriers to the introduction/opposition to new role
· Impact/defining and measuring success
· Development opportunities for the role/Career development opportunities
· Service user perspectives on the role

One researcher (LW) was responsible for the indexing of themes into a framework and initial mapping and interpreting of those themes. Analysis meetings were held between AM and LW that included systematic data verification and challenging of interpretive analysis. Independent verification of emergent themes was undertaken by a senior health services research academic (SN). The information presented in this manuscript was informed and verified by two tiers of the health service executive and the podiatry manager (KM).

Ethics approval was obtained through the ACT Health Directorate Human Research Ethics Committee (ref ETHLR.11.038). Due to the small number of participants, some verbatim quotes have not been labelled to protect participant identity.

### Findings

#### Why the role was introduced

The podiatry assistant role was initiated by managers with a request for funding targeted at workforce initiatives to address *difficulties in retention and recruitment…* It was agreed that implementation of a podiatry assistant could have ‘*multi-faceted potential benefits’,* specifically that while there was an existing foot-care kind of nursing service in place,*’ it seemed to make much more sense to place the foot-care clinics under the podiatry team’.* Furthermore, the proposal made sense from a workload perspective as podiatry

‘.was in the position of being swamped with a lot of clients and needed to just see the high risk clients. And this was a way to cope with that workload’. [allied health managers]

The podiatry manager stated that one of the issues regarding the nurses delivering basic foot-care *‘is those nurses aren’t part of the podiatry team’.* The allied health managers identified that continuity of service was compromised as ‘*clients probably didn’t get a great benefit from having that disjointed service’.* Lack of continuity emerged as an issue when dealing with clinical governance issues. As the podiatrists reported

… there were so many clinical governance issues around the foot-care service. I think it was always this really grey area around who was actually responsible for that service, was it the podiatrists who were referring it, or was it the nurses who were basically the managers of these FCNs. I think they saw an advantage to bring it under the podiatry umbrella, so that we could actually govern it. [podiatrist]

#### Roles and Responsibilities

##### Scope of practice

Managers, professionals and the podiatry assistant all reported that an important component for successful implementation of the PA role was the development of a policy specific to the role and scope of the PA (Tables 3 and 4). Allied health managers saw this as a particularly important risk management strategy for the podiatry staff who would be supervising the PA because PAs are not registered. Some podiatrists had initially requested that the PA should be directly supervised by a podiatrist but acknowledged that a direct supervision model ‘*where you had somebody standing over your shoulder just doesn’t work’ [manager].* Documenting the PA’s scope of practice provided a clear definition of the role, therefore instilling a level confidence in the PA role, enabling a more ‘hands off’ supervision model*.*

**Table 3:** Scope of practice policy regarding tasks not to be undertaken by the assistant (p2)

**The following tasks must not be included in a podiatry assistant’s scope of practice under any circumstances:**	
a)	Clinical interpretation of podiatry referrals
b)	Interpretation of a patient’s diagnosis or prognosis
c)	Interpretation of assessment findings
d)	Development of a physical diagnosis
e)	Development or modification of a patient’s treatment or care plan
f)	Clinical advice outside the parameters of an approved care plan or standardised general health promotion information
g)	Casting for custom orthoses
h)	Moulding of prefabricated or non-casted mouldable orthoses
i)	Clinical evaluation or review of treatment modalities
j)	Clinical treatment involving the use of a scalpel
k)	Injections and/or surgical procedures
l) Clinical assessment or examination of patients including:	
-Biomechanical(including postural or gait assessments)	
-Muscular	
-Orthopaedic	
-Neurological	
-Vascular	
-Dermatological	

**Table 4:** Scope of practice policy regarding registered podiatrist responsibilities (p2-3)

**Registered Podiatrist**	
A registered podiatrist is at all times professionally responsible for a patient’s care plan and treatment, the podiatry assistant, their scope of practice and conduct whilst being directly accountable for the care a patient receives. The podiatrist must be familiar with and work within the Podiatry Board of Australia Guidelines for podiatrists working with podiatric assistants in podiatry practice.	
The registered podiatrist must ensure the following:	
a)	All patients have an initial assessment completed and an appropriate care plan recorded prior to involving an assistant in any treatment.
	The care plan clearly delineates the treatment that will fall within the appropriate scope of practice for the assistant.
b)	The patient is reassessed and their care plan renewed annually.
c)	The podiatry assistant has the required minimum qualifications, training and competencies to complete the delegated tasks.
d)	A podiatry assistant is at no time delegated tasks outside their scope of practice or for which they have not yet been deemed competent or not safe to perform particularly when they undertaking training.
e)	All warning and safety procedures are undertaken with patients including instructions regarding contraindications, adverse reactions and expected reactions of the techniques to be applied.
f)	The podiatry assistant fully understands and can implement the process for reporting both clinical and non-clinical emergencies.
g)	Where a podiatry assistant is working without direct supervision and a clinical emergency occurs documented protocols exist that specify the process for reporting unexpected changes to a patient’s health or foot care needs.
h)	A podiatry assistant is at all times clearly and correctly identified as an assistant. A referral may only occur once discussed with a patient and informed consent obtained.
i)	A podiatry assistant is provided with clear directions of the work to be undertaken.
j)	Clearly defined lines of communication and direction are established and the assistant is aware of these.
k)	The quality of work completed by the assistant is regularly evaluated to ensure they are working in a competent and safe manner.

##### Clinical Governance

Having a clear scope of practice was seen as important from a governance point of view, due to a certain level of ‘*nervousness about what if this person does something wrong’* [managers]. This also clarified the implications if the PA practiced beyond the scope of practice. The allied health managers suggested that the assistant’s scope of practice needed clarification

‘to ensure the team were aware of the implications for them if the assistant was to step outside her scope of practice, despite having that policy in place, it’s, “Am I going to get in trouble for that”.’ [allied health manager]

The need for a clear and understood scope of practice policy was highlighted by the podiatrists who talked about managing the PA’s ‘*enthusiasm’* and initial tendency to *‘overstep the mark’.* This was addressed through developing the scope of practice document, ‘*using language that was very clear and defined’* and embedding the scope of practice in workbooks, assessments and manuals and by staff iterating scope of practice with the PA.

As demonstrated in this exchange, the podiatry assistant demonstrated an applied knowledge of their scope of practice and expressed confidence in remaining within it.

Interviewer: Do you feel that the patients that end up in your care are appropriate?

PA: Yeah, I do get some that I have a bit of a query about. Like their nails might be a little bit too thick, and then I will talk to the podiatrist and then we’ll see. I can refer back to the podiatrist. They don’t have to stay in my care if I don’t think that I can treat them.

The PA went on to express that the boundaries imposed by the scope of practice contributed to her feeling effective in her role.

##### Developing and defining scope of practice

National and state policies were important in defining the scope of practice of the PA [25,26]. For example the ACT Podiatrist’s Board states ‘restricted duties include treatment involving the use of a scalpel’. As such this requirement, along with other tasks not to be undertaken by the assistant, were included in the scope of practice document, Table 3[25,26]. This was clearly understood by the PA.

‘*I’m not allowed to use a scalpel. Usually I just file, and use the nail clippers. And that is the range of what I am allowed to do.’ [PA]*

From the perspective of both the allied health managers and the podiatry assistant the scope of practice was largely determined by risk level of the patients The level of risk was determined by the referral and screening process as all referrals to the PA came from the podiatrist who had assessed the client as being low risk and made judgements about case complexity. The scope of practice document clearly outlines the responsibilities of the registered podiatrist in ensuring patients have had an initial assessment that *‘delineates the treatment that will fall within the appropriate scope of practice for the assistant’* (Table 3). The importance of having confidence in the podiatrists making the right decisions regarding referrals was reiterated by the PA.

‘.*.the podiatrists are quite thorough and make sure that I am not getting complex cases….I guess they know my scope of practice.’ [PA]*

There was a clear understanding of the relationship between the risk level of the patient and the PA’s scope of practice as expressed here:

‘…*my scope of practice is um dealing with the lower risk patients. People who can’t really reach their feet….a lot of them do have diabetes and I guess it’s a difficult thing if they start, you know, cutting their own nails. Then they’ve got more risk of cutting themselves and then causing infections….people with eyesight problems..so I just deal with lower risk patients.’ [PA]*

Service users reiterated the level of risk as an important indicator of their satisfaction with seeing the PA instead of the qualified podiatrist*:*

*‘I am quite happy to have an assistant, because I only need my nails cut anyway, there’s no big dark secret. So yeah I am very happy*…’*[service user]*

#### Supervision and support structures

Initially, full supervision was provided by the podiatry clinical educator and principal supervising podiatrist whose responsibility was to provide the assistant’s training and supervise the assistant in completing her competencies. This supervision gradually reduced as the assistant gained experience. Even at this stage, however the PA found it reassuring to have the podiatrist on hand and described the importance of the on-going support and supervision.

Reflective practice was an important component of supervision, including providing honest and constructive feedback as a supervision strategy. However some podiatrists were uncomfortable providing critical feedback to the assistant because the assistant was seen as being a member of their team as opposed to a student who will leave the team at the end of their placement.

‘*Even though the podiatrists had had [podiatry] students, they didn’t want to discourage her [the assistant] because she was new and she was training and the whole thing was new and exciting. They didn’t really want to give that negative feedback’. [allied health manager]*

The challenge of providing feedback was compounded by the unknown skill levels and expectations of the PA at such an early stage in the implementation of the new role.

‘*I think that was part of the challenge, having to get the primary supervisors to just see that they are not just here to supervise the job, but to say what you can improve on as well. I think that took a little bit of getting used to.’ [podiatrist]*

Unique to the traineeship model was the collaboration and negotiation between the Registered Training Organisation (RTO) and the workplace, however this brought with it conflicting supervision and management approaches and expectations. This was particularly evident in the workplace where multiple local podiatrists supervised the PA at different times during the traineeship.

‘*Different supervisors have differences as well… I think trainees are looking for one consistent way of dealing with some of those issues. And we don’t have one consistent way.’ [podiatrist]*

#### Negotiation of roles

Historically nursing staff performed the foot-care role, and the transfer of the responsibility to the podiatry service has involved on-going negotiation between nursing and podiatry management.

‘*We initially started to do foot-care as a favour to podiatry because they were short staffed. And then it kind of got set as a precedent so we never quite got out of that.*’ *[nurse manager]*

The podiatrists sensed that the nursing team attached a stigma to the foot-care role. This tension was also identified by a service user who had seen the PA after having seen the foot-care nurse in the past.

*‘The foot-care nurse said to me “No I’m not paid to be the podiatrist”. I said alright, that’s fine*.’ *[service user]*

The foot-care nurses (FCNs) said that they were told they had to take on the role due to high unmet demand for podiatry and long waiting lists*.* Some of the foot-care nurses talked about their enjoyment of the role and feeling ‘*used’* after completing ten years of experience in the foot-care service.

‘*Now they’re taking it away from us after 10 years and you kind of feel a bit used* …*We’ve really enjoyed it and got a good rapport with some of them that come in to us. For years and years and years, and we really enjoyed it. And a lot of the people don’t enjoy foot-care but we enjoy it.’ [FCN]*

The allied health and nursing managers were in agreement that it made sense to replace the FCNs with a PA who would be part of the podiatry team. The allied health managers wanted the PA to ensure clinical expertise and continuity of service remained within the podiatry service. The nursing managers wanted to reduce the burden on the nurses of completing the foot-care role in addition to their regular nursing tasks.

The foot-care nurses were not involved in the initial consultation process regarding their role and the podiatrists highlighted that communication between the foot-care nurses and podiatrists had been limited in the past.

‘The podiatrists would leave and the foot-care nurses came in to use the same space. So there wasn’t really much communication about patients and things like that.’ [allied health manager]

While the necessity of the PA role was unanimous, there was some tension when the foot-care nurses were asked to provide the foot-care service when the PA was not available, although this only occurred during a period of extended leave.

#### The traineeship model

The flexibility of the traineeship model was appealing to the managers and this fitted with the purpose of the targeted workforce initiative funding. The opportunity to capture the interest of potential employees who may not necessarily possess a specific skill set was also important.

The traineeship model was perceived to have the advantage that an assistant could be trained more quickly. However this was problematic due to delays in recruitment which meant that the trainee was unable to undertake the first session of formal training. Therefore the podiatry service was unprepared for a trainee who had no generic healthcare skills when they started and needed to immediately train them in the skills required to work in a clinical setting.

The traineeship model also offered the opportunity for a podiatry assistant to enter the workforce without having completed the Certificate IV with gradual exposure to the podiatry profession in parallel with more formal study. It allowed the implementation of the role to be managed with greater flexibility in terms of how the podiatry assistant developed their skills and knowledge. The allied health managers identified this flexibility as a downside and benefit*,* whereby the flexibility over the way competencies were developed and implemented in the workplace was at times offset by the responsibility of ensuring the trainee received the necessary education.

Although an agreement (between the RTO and ACT Health) was reached to deliver the podiatry-specific skills in a traineeship, thus allowing for access to appropriate resources and specialist clinician skills in a work based learning model that the RTO could not otherwise provide, the traineeship model required extensive investment in time and training by the podiatrists. One suggestion by the podiatrists for future development for the Certificate IV was for the trainee to have generic health care skills training through the RTO followed by intensive, discipline specific theory block sections for each competency delivered by the workplace which is then followed by observation and practical.

#### Impact of the new role

The impact of the PA role had not been measured at the time of the evaluation.

‘*I mean most people’s gut feelings to me is that the roles are working. But in terms of an evidence base to hang your hat on and say and there you go, it is working, I don’t think we are at that point.’ [allied health manager]*

It was expressed that measuring the impact of the introduction of the role on the podiatry service would be difficult for a number of reasons. First due to the length of time the PA would be technically qualified and therefore able to demonstrate an impact:

‘*it’s probably 6–12 months down the track at least before we could realistically assess the effectiveness of those people in those roles*.’ *[allied health manager]*

And second, they would need to measure the impact on nursing rather than podiatry to

‘*look to see if it actually increased the occasions of service (number of treatments) for nursing for their own patients. Freeing up their staff [nursing]. Really that’s the only way we could see of potentially measuring it’. [podiatrist]*

This was keenly recognised by those interviewed. As one podiatrist exclaimed, ‘*I don’t think she actually has. I don’t think it actually impacts on our workload.’*

##### Perceived impact of the role

Managers and podiatrists reported that the act of transferring low risk foot-care from FCNs to the PA in two of four geographic localities meant that although the number of podiatry treatments increased, service capacity, in the form of freeing up of podiatrist’s time was unchanged. It was recognised, however, by all participants that there was a need for greater capacity, potentially in the form of more PAs, to transfer the responsibility of *all* foot-care clinics across to the podiatry service. This would enable realisation of the full potential of the PA role.

However several other important outcomes were identified. Podiatrists summarised the impact of the role as improving their relationship with nurses and the nursing team and having control over the PA role and the benefit of;

‘*having someone who actually seems enthusiastic and enjoys her role rather than being forced to do something that they don’t want to do’. [podiatrist]*

This was despite several of the FCN’s expressing their disappointment at no longer performing the PA role.

There was also a perception that the PA role improved communication processes between clients and the podiatry service because the PA was part of the podiatry team. Having the PA as part of the podiatry team, being supervised by podiatrists, was recognised as an important part of clinical governance processes and thus the foot-care service could be more effectively and safely managed.

‘*The two big impacts for me is that potentially we can take over the foot-care nurses so allowing them to go back to their role and to do their thing. Um, allowing us to have better governance over the service, or the foot-care service.’ [podiatrist]*

Managers and podiatrists also discussed that regaining control over the foot-care service through the use of a PA would go some way to assist smoother patient pathways through the podiatry service, although there was no empirical evidence to support this.

A key difference identified by all participants between the PA and the FCNs is the amount of theory that has been undertaken to perform the role. It was identified that the immediate impact of such subtle differences in training and approach to care is difficult to measure. The PA herself however commented that having good knowledge of foot conditions helped her to be proactive with foot-care:

‘*Knowing, having the knowledge of feet conditions. Because sometimes you might actually pick up on something. Say, you know they have never had moles on their feet, or something like that, and no-one has actually reported that in the notes, and obviously one day a mole has appeared on their foot, well then you sort of haven’t…you sort of…I guess you think to yourself, well that wasn’t there, and now it’s here, well you should go to your GP and get that checked out’. [PA]*

There was also discussion that in the future there may be capacity for the role to demonstrate an impact on podiatrist time with the PA’s ‘*ability to help with podiatric surgery. Which that at the moment requires two podiatrists’. [podiatrist]*

Overall, staff and service users expressed high levels of satisfaction with the PA. One service user related:

‘*She had more to spend with me and she did small things for me and she actually had time to do it. Because when I saw [her] she told me to use the file and that and when I told the nurse, she said “No I’m not paid to be the podiatrist”. I said alright, that’s fine, but the girl I have she was really terrific.’ [service user]*

A further success of the traineeship approach was the high level of satisfaction the trainee reported with the role, and the traineeship process, and the level of satisfaction expressed by managers and podiatrists who worked with the trainee.

#### Facilitators and barriers to successful implementation

##### Facilitators

The engagement of key staff, particularly the podiatry staff, managers and the executive level allied health advisor was seen as key to success. The recognition of the mutually beneficial nature of the new role also facilitated the implementation of the role.

The allied health managers and podiatrists identified that having an allied health advisor as a conduit for information between the RTO and the workplace was essential to the introduction of the PA role. Similarly the podiatrists identified that the support of the allied health advisor was integral to the process.

The personal attributes of the PA facilitated successful implementation of the role from both staff and service user perspectives. The PA demonstrated maturity and an enthusiasm for caring for people and a tangible enjoyment from the role. The skills and attributes required by the PA included communication (English), ability to relate to people, good written skills, interested in people, caring and hand-eye coordination skills.

The role and personal attributes of the clinical educator, who is trained in delivering education and supervision to clinical staff, was also perceived as an essential facilitator of the role development.

Targeted recruitment was key to successful implementation. The allied health managers and podiatrists expressed the importance of advertising specifically for a podiatry trainee rather than a generic allied health trainee, as it was felt that the trainee needed to have an interest in or knowledge of what it may be like to work with feet.

##### Barriers

Organisational culture: Organisational culture was particularly complex because the implementation of the PA role involved managing the cultures of two different organisations (the RTO and the health service).

Employment classification: A delay in recruiting the trainee due to classification issues under the enterprise bargaining agreement (agreements made between employers and employees about terms and conditions of employment) placed the trainee and workplace under pressure to provide initial training that was unable to be undertaken at the RTO as academic teaching sessions began before recruitment was finalised.

Timing with respect to training cycle: Poor timing was identified as one of the primary barriers to the implementation of the PA role. The delays in classifying the role and recruiting the trainee meant that by the time they started, the beginning of the year at the RTO had already commenced.

‘…they couldn’t pick up the subjects that they had missed because of their late start date, we had to look around to see what other learning opportunities that were out there that we could sign them off’ [manager].

The timing of the introduction of the role meant that there was little time for extended planning and collaboration between all key stakeholders to collectively provide input into the development of the role, training needs of staff and the training package itself.

Lack of training capacity and resources to support national competencies: The ‘Certificate IV’ AHA competencies are part of a health training package, developed at a national level with input from the Industry Skills council and consultation with industry experts. To be awarded the Certificate IV AHA (podiatry) there is a requirement to satisfy the four specific competencies as part of the podiatry skill set (Tables 5 &6) as well as 18 other more general competencies, such as ‘HLTCSD305B Assist with client movement’.

**Table 5:** Podiatry specific skill sets or competencies for the certificate IV AHA

**HLTIN302A**	**Process reusable instruments and equipment in health work**
HLTAH406A	Assist with podiatry assessment and exercise
HLTAH405A	Assist with podiatric procedures
HLTAH404A	Assist with basic foot hygiene

**Table 6:** Performance criteria for certificate IV AHA in podiatry to perform basic foot hygiene (page 2, health training package, HLTAHA404A assist with basic foot hygiene)

2.1	Explain to the client the purpose, rationale and requirements of the foot hygiene session
2.2	Determine the client’s understanding of the purpose, rationale and requirements of each part of the foot hygiene session
2.3	Identify any *condition indicating the client is at high risk* that requires podiatrist attention
2.4	Assist client in and out of shoes, socks and hosiery where necessary
2.5	Correctly position the client prior to foot hygiene session
2.6	Implement necessary infection control measures
2.7	Perform basic foot hygiene according to the directions of the podiatrist and using appropriate infection control precautions, especially in relation to air borne particles
2.8	Apply appropriate *dressings* to any skin breaks which might result from treatment
2.9	Provide feedback that reinforces the podiatrist’s advice
2.10	Identify and manage *client compliance* issues.
2.11	Work with client to determine and plan any follow up requirements and dates
2.12	Seek assistance when client presents with needs or signs outside limits of own authority
2.13	Report client difficulties to the supervising podiatrist

Lack of capacity for training by the RTO was however identified by participants as a barrier to implementing the role. It was recognised from the outset that it would be difficult for the RTO to provide training given the small numbers pursuing podiatry specific qualifications. Specialised skills required for podiatry training meant unique training requirements could not easily be met within the RTO’s resources and framework, which has been created around physiotherapy and occupational therapy training.

The lack of supporting resources from the RTO meant that the podiatrists felt the need to undertake significant development of learning resources and work based learning activities to ensure national competencies were met. The majority of this responsibility fell on the shoulders of the clinical educator who was employed in a 0.5 FTE position. As expressed by the podiatrists,

‘*if the traineeship thing was a regular thing, I would say that this position is to be full time to be able to provide that type of training appropriately*.’

These issues were partially resolved further into the traineeship when a competency resource for PAs, developed to support the national competencies, was identified and purchased from an organisation external to the RTO.

Lack of skills to implement training: These issues were compounded by a lack of experience and knowledge of how to work with *‘someone with no knowledge and no background’* as identified by the podiatrists. Training and assessing competence for university students was at a very different level to that for a trainee assistant.

Additional training demand on podiatrists: Furthermore, the podiatrists felt that the traineeship ‘*was fairly full on’* because the PA shadowed their clinical practice which in the initial stages created extra demand on the podiatrists as they were *‘trying to explain various things but not say this is your role, and trying to not make it boring’.*

## Discussion

Overall, the implementation of PA traineeship was perceived as successful in terms of the transfer of responsibility for the basic foot-care service from nursing to podiatry, and high levels of staff, trainee and service user satisfaction with the role. Although not quantified, it was perceived that the two areas most impacted upon by introducing the PA role were nursing capacity, by releasing ENs from foot-care duties thus redirecting EN hours back to core nursing duties and an increase in the number of treatments delivered by the podiatry service. Although it should be pointed out that this was not an overall net gain in treatment capacity, rather, a shift in treatment provision away from nursing to podiatry. The transfer of the service back to podiatry responsibility also had a perceived impact on communication and feedback loops between the service provider (PA) and the podiatry service, the nursing-podiatry relationship, clinical governance around the foot-care service and continuity of care for clients through the podiatry service.

For these outcomes to be enhanced, and for there to be a greater impact on podiatry service capacity, there needs to be more capacity within the PA role potentially through employment of more PAs. This would enable the transfer of all low risk foot-care to the podiatry service and enable the podiatry service to utilise the PA role for other duties such as assisting with surgery.

This evaluation suggests that employing a PA within an appropriate training and supervision support framework is a very low risk approach to subsuming aspects of podiatry work traditionally undertaken by other health care providers. It also facilitates a more seamless patient journey by enabling podiatrists to co-ordinate the full range of foot treatment services

These findings go some way to demonstrate a means to maintain a monopoly over key aspects of podiatry work and minimise the profession’s vulnerability to boundary encroachment by other professions as suggested by Borthwick et al. [27]. There is a need however to more formally evaluate the relationship between reclaiming podiatry work and the impact this has on the service user.

The challenge to this argument for a professional monopoly around the lower limb is that the low-risk foot care service had been provided successfully by the FCN for nearly a decade. This meant that podiatrists did not manage the foot problems of lower risk clients, however, nor did the demand for their services decline over this period. Additionally, where the PA will be restricted to working solely in a podiatric role, the ability of the enrolled nurses to backfill for the PA, then return to other duties highlights their value to the service in terms of their flexibility and broad range of skills. In this sense, the FCN role is more akin to a generic practitioner.

This research has demonstrated that successful implementation of a PA role was perceived to be dependent on clear and rigid role boundaries, defined by a documented scope of practice. However greater flexibility and autonomy in support worker roles has been shown to significantly improve support worker retention and career progression opportunities [28]. Furthermore it has been shown that greater flexibility of roles and responsibilities for support staff enables these workers to better fill local gaps in service delivery. Therefore there is a need to recognise that a balance may need to be achieved between defining clear role boundaries and embedding flexibility and autonomy into a support role.

The important contribution of this model has been the traineeship approach. The traineeship was deemed to be successful in terms of producing a PA whose skills were shaped by, and directly met the needs of the practitioners with whom she works. The model gave podiatry staff the opportunity for reflection on what was working and what was not. This combined with the flexibility to change things based on reflection strengthened the confidence the staff had in the PA’s competence.

The model of engaging the supervising podiatrists in the development and implementation of the role is an important strength of this approach. In contrast to previous examples of assistant implementation, the supervising podiatrists had a clear understanding of the PA role, ability and competence, and as such had confidence to delegate work to the PA.

However, the high resource intensity of the traineeship model was acknowledged by most programme participants. It took 18 months before the trainee was practicing to their full scope of practice and required extensive input from the supervising and support staff. Staff perceived that the role could be deployed more rapidly if the formal training was undertaken in an intensive block, followed by the in-house training. As the competencies and training framework have now been established within the podiatry team, this approach is likely to be effective should a traineeship be offered again.

There was a clear perception that the lack of RTO facilities to provide the podiatry specific aspects of the training (both clinical and theory elements) to meet nationally developed podiatry specific competencies, meant that the burden of delivering the traineeship fell primarily on the workplace. Despite what was perceived as good communication and relationships with the RTO, further discussion between the RTO and the podiatry team may have been useful to further clarify roles and responsibilities and to determine a more balanced input into the traineeship. Furthermore, there is a need for better sharing of resources for implementing new assistant roles. One example is a resource sharing community that has been developed for those who utilise the Calderdale Framework to implement new assistant roles [29]. Adherence to the domains of the Calderdale Framework has also been associated with better sustainability, more efficient use and greater career development opportunities of new assistant roles [30].

Subsequent to the evaluation, the podiatry manager reported that the trainee undertook competency based training in nail surgery and orthotic modification. The impact of this has been a significant release of podiatrist time as there is now one podiatrist and one PA performing surgery instead of two podiatrists and orthotic modification/fabrication is being undertaken by the PA following a prescription from the treating podiatrist.

### Study limitations

This study examined the implementation of one new assistant role that was yet to demonstrate its full potential. As such the conclusions that can be drawn from this study regarding the impact the role has had are limited. This is however the first evaluation of the implementation of a new PA role in Australia that has utilised a unique traineeship approach. The learning from this study adds to a growing body of research examining mechanisms for successful workforce change [30,31].

## Conclusions

The AHA Traineeship model developed by the ACT Government Health Directorate is an innovative and effective way of implementing a new role in a health care setting. There is no doubt that the new role has been effective in increasing nursing capacity and increasing occasions of service for podiatry and there seems to be added benefits of the new role to the podiatry service in terms of regaining control over podiatric services and as such, improving clinical governance and patient pathways. Further exploration is required to examine the impact these outcomes have on service users and developing capacity among podiatrists. This research has demonstrated that the implementation of a PA role using a traineeship approach requires good coordination and communication with a number of agencies and staff and substantial resources to support training and supervision.

## Competing interests

Kerryn Maher is the director of the podiatry service which was the subject of this evaluation. She was not involved in the data collection or interpretation, but guided the research questions. There are no other competing interests.

## Authors’ contributions

AM secured the funding to undertake the evaluation and led the evaluation; SN, RB and AB were co-applicants on the funding application, guided the research questions and helped draft the manuscript. LW was involved in the data collection, analysis and preparation of the paper. KM provided service access, helped coordinate the interviews and focus groups, and helped draft and comment on the manuscript. All authors read and approved the final manuscript.
